# Closing the Wearable Gap—Part II: Sensor Orientation and Placement for Foot and Ankle Joint Kinematic Measurements

**DOI:** 10.3390/s19163509

**Published:** 2019-08-10

**Authors:** David Saucier, Tony Luczak, Phuoc Nguyen, Samaneh Davarzani, Preston Peranich, John E. Ball, Reuben F. Burch, Brian K. Smith, Harish Chander, Adam Knight, R. K. Prabhu

**Affiliations:** 1 Electrical and Computer Engineering, Mississippi State University, Mississippi State, MS 39762, USA; 2 Industrial and Systems Engineering, Mississippi State University, Mississippi State, MS 39762, USA; 3 Kinesiology, Mississippi State University, Mississippi State, MS 39762, USA; 4 Agricultural and Biological Engineering, Mississippi State University, Mississippi State, MS 39762, USA

**Keywords:** soft robotic sensors, wearables, athletic training, ankle complex, plantar flexion, dorsiflexion, inversion, eversion, sensor placement, kinematic

## Abstract

The linearity of soft robotic sensors (SRS) was recently validated for movement angle assessment using a rigid body structure that accurately depicted critical movements of the foot–ankle complex. The purpose of this study was to continue the validation of SRS for joint angle movement capture on 10 participants (five male and five female) performing ankle movements in a non-weight bearing, high-seated, sitting position. The four basic ankle movements—plantar flexion (PF), dorsiflexion (DF), inversion (INV), and eversion (EVR)—were assessed individually in order to select good placement and orientation configurations (POCs) for four SRS positioned to capture each movement type. PF, INV, and EVR each had three POCs identified based on bony landmarks of the foot and ankle while the DF location was only tested for one POC. Each participant wore a specialized compression sock where the SRS could be consistently tested from all POCs for each participant. The movement data collected from each sensor was then compared against 3D motion capture data. *R*-squared and root-mean-squared error averages were used to assess relative and absolute measures of fit to motion capture output. Participant robustness, opposing movements, and gender were also used to identify good SRS POC placement for foot–ankle movement capture.

## 1. Introduction

Biomechanical analyses of human joint range of motion (ROM) have evolved from simple goniometric measures to technologically advanced optical motion capture systems. While motion capture technology aids in the assessment of joint range of motion with gold standard precision measures [1], the use of this technology is primarily confined within a laboratory setting, with limited applicability to changes in joint angles that occur in everyday tasks. Moreover, the financial cost and time consumed are also greater with the use of laboratory-based motion capture equipment. Therefore, there is great demand for alternative solutions to precisely measure joint kinematics outside of a laboratory that have lower financial and time cost requirements and can capture day-to-day real-life scenarios. A wearable device that can measure changes in joint range of motion and limit the negative aspects of motion capture while being precise is a potentially promising solution [2].

Very recently, soft robotic sensors (SRS) (liquid metal sensors) that change in resistance when stretched were used to determine if ankle joint movements could be inferred by using a custom-built, rigid-body ankle joint mechanical device [3]. This device mimicked the ankle joint in its ability to perform ankle joint motions of plantar flexion, inversion and eversion. A single sensor was placed on the middle of the ankle joint device and all ankle movements were performed passively on the device with data from the sensor recorded for each ankle movement at various degrees of ROM quantified by using an electric goniometer and two smart phones with the RIDGID level iOS application installed [3]. Based on the results of this study, the sensor could provide significant linear models in predicting ankle joint plantar flexion. However, the inversion and eversion movements were still difficult to predict accurately with only a single sensor being used. It was concluded that there was a significant linear relationship between the positional change of the mechanical device and the resistance values of the soft robotic sensor for the ankle plantar flexion movement. This indicated that the sensor was promising to be a potential “out-of-the-lab” substitute to inertial measurement units (IMU) and motion capture based solutions to quantify human joint kinematics [3]. Utilizing SRS offers the advantage of contouring over the anatomical surface of the human joints, thus mitigating the drift in measurements associated with IMUs. Furthermore, they can be embedded into garments to function as a wearable solution [4,5,6].

While SRS were found to be successfully indicative of ankle ROM during plantar flexion, inversion and eversion, these were still conducted on a mechanical ankle joint device that is far from animate and real-life, compared to the human foot–ankle complex. The human ankle and foot—unlike the mechanical device—has intrinsic properties of living tissues such as skeletal muscles that are responsible for force production to cause joint rotation and bones that act as attachment sites for muscles and as mechanical levers for human movement [7,8]. One of the major limitations outlined in the previous study was that the ankle joint complex has three-dimensional movement and thus three degrees of freedom (DOF), with varied ROM for each DOF [3]. More importantly, these DOF rarely occur in isolation in a real-world setting or during activities of daily living but occur as a combination with other DOF of the ankle and foot segment [7,8]. It was also reported that a single SRS can detect plantar flexion, inversion and eversion, but it was not capable of differentiating inversion and eversion [3], subsequently mandating the need for testing human participants with active movements of the ankle and foot segments. Additionally, the SRS placement and orientation on human participants have not been analyzed yet, which can present additional challenges in data collection and interpretation. Finally, the SRS data used to infer ankle and foot movements need to be validated against the state-of-the-art technology using motion capture analyses. Therefore, the purpose of the study was to analyze various SRS placements and orientations when using more than one SRS, to determine good positional and surface anatomical location of the SRS on the foot–ankle segment that provides the most accurate and precise ankle and foot ROM measurements validated against a 3D motion capture system. The primary contributions of this paper are:

1. The initial work in [3] was extended to human movement.
2. SRS sensor placement was analyzed and optimal sensor placement was determined for plantar flexion (PF), dorsiflexion (DF), inversion (INV), and eversion (EVR).
3. Linear model analysis results show high levels of goodness-of-fit and low RMSE values, meaning the proposed placements are effective at measuring INV, EVR, PF and DF compared to the gold-standard motion capture solution.

## 2. Materials and Methods

### 2.1. Participants

Ten participants (five males: age, 22–24; height, 170–193 cm; mass, 71–100 kg; foot size, 10–13; and five females: age, 23–25; height, 156–168 cm; weight, 48–66 kg; foot size, 5.5–8) with no history of lower extremity musculoskeletal injuries/surgeries or neuromuscular diseases/disorders were tested. This sample was chosen to test both genders and individuals with small, medium, large and extra-large sized feet. Since this was a preliminary study, the sample size was set at 10 participants, which is consistent with a recent study that examined gait kinematics of the foot and ankle among 10 participants [9]. All participants read and signed an informed consent to participate in the study. The study was approved for human subjects testing by the University’s Institutional Review Board (IRB).

### 2.2. Study Design

The study design followed a single day testing protocol with an initial familiarization session that was conducted prior to the experimental testing. All participants visited the Human Performance Lab (HPL) at the University’s Center for Advanced Vehicular Systems (CAVS) research center. During familiarization, all participants were briefed on the procedures and provided an opportunity to perform a few trials of the experiment that included simple ankle movements in a non-weight bearing (NWB), high-seated, sitting position (explained further under experimental procedures). Following the familiarization process, the participants performed the experimental testing.

### 2.3. Instrumentation and Participant Preparation

The experimental testing included measurements of ankle joint kinematics using 12 Bonita 10 camera 3D motion capture system (Vicon, Oxford, UK). The SRS used in the current study included the StretchSense sensors (Auckland, New Zealand). The motion capture data were sampled at 100 Hz and the SRS data were sampled at 25 Hz. For this study, only the right foot–ankle complex was measured. During testing, each participant was prepared by placing reflective motion capture marker clusters on the right lower extremity for the foot and shank (lower leg) segments. Four SRS were placed on the ankle and foot segment in a predetermined placement and orientation configuration (POC), explained further in Section 2.4.1.

### 2.4. Movements

**Figure 1:**
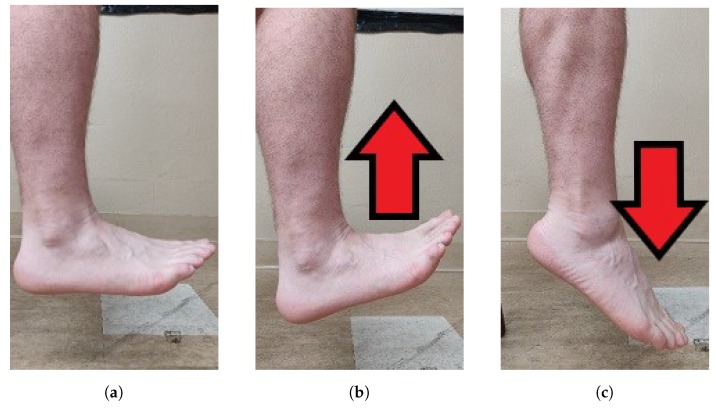
Sagittal plane: (**a**) neutral; (**b**) dorsiflexion; and (**c**) plantar flexion.

**Figure 2:**
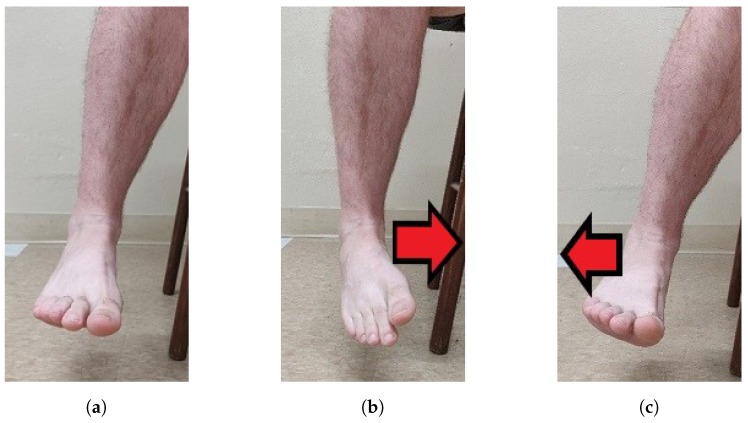
Frontal plane: (**a**) neutral; (**b**) inversion; and (**c**) eversion.

The participants started in neutral positions. Figure 1 shows the neutral, DF and PF movements in the sagittal plane, and Figure 2 shows the neutral, INV and EVR movements in the frontal plane.

#### 2.4.1. SRS POCs

Three different POCs were determined based on bony landmarks and movement patterns of the foot–ankle segment. The individual SRS were then labeled based on the movement they were positioned and oriented to measure. The sensors were located in a position that would cause them to be stretched by the corresponding joint motion. The PF SRS was mounted on the dorsal surface of the foot to measure the downward movement of the foot, such as when the toes are pointed towards the ground and the angle between the dorsal surface of the foot and the lower leg increases [7,8]. The INV SRS was mounted on the lateral side of the ankle to measure the movement of the sole (bottom of the foot) towards the midline of the body [7,8]. The DF SRS was mounted on the heel of the foot to measure the upward movement of the foot towards the lower leg (angle between the top of the foot and lower leg increases) [7,8]. The EVR SRS was mounted on the medial side of the ankle to measure the movement of the sole away from the midline of the body [7,8].

**Figure 3:**
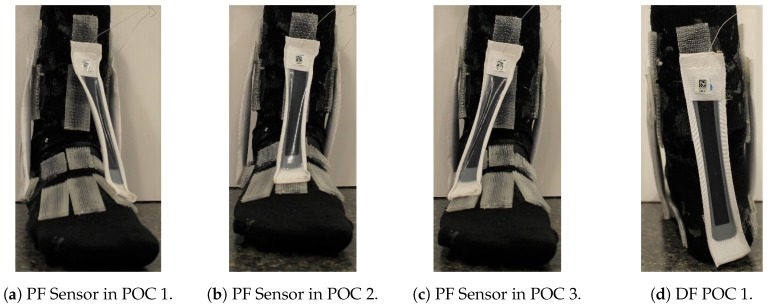
PF and DF sensor POC positions: (**a**) POC 1; (**b**) POC 2; (**c**) POC 3; and (**d**) The DF sensor was positioned the same for all three POCs (heel of the foot is pictured).

**Figure 4:**
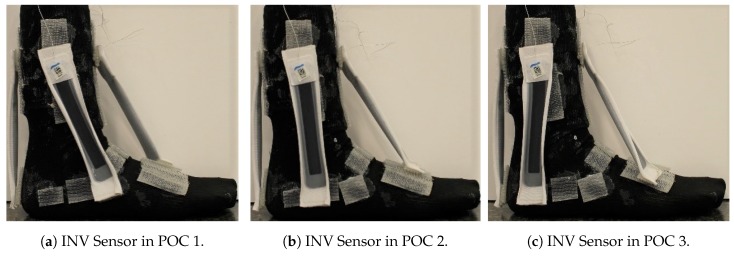
INV Sensor POC Positions: (**a**) POC 1; (**b**) POC 2; and (**c**) POC 3.

**Figure 5:**
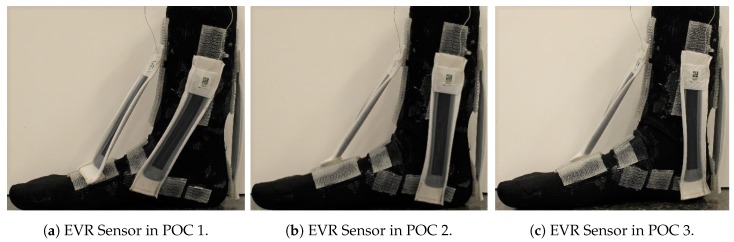
EVR sensor POC positions: (**a**) POC 1; (**b**) POC 2; and (**c**) POC 3.

The PF POCs were determined based on the hallux (big toe) and surface of the top of the foot. The PF SRS was first oriented towards the hallux, then over the middle of the foot, and lastly towards the 5th phalanx. The INV POCs were centered around the lateral malleolus (bony landmark on the lateral side of the ankle). The INV SRS was first positioned anterior to the lateral malleolus, near to the 5th phalanx, then directly over the lateral malleolus, and finally, posterior to the lateral malleolus, close to the heel of the foot. The DF SRS was kept in the same position over the heel for all three POCs, due to the small surface area in which the DF movement could be measured. The POCs for the EVR SRS were determined similarly to the INV POCs, except they were based on the medial malleolus (bony landmark on the medial of the ankle). For the first and third POCs, the EVR SRS was kept parallel with the INV SRS. These POCs are illustrated in Figure 3, Figure 4 and Figure 5.

### 2.5. Experimental Procedures

**Figure 6:**
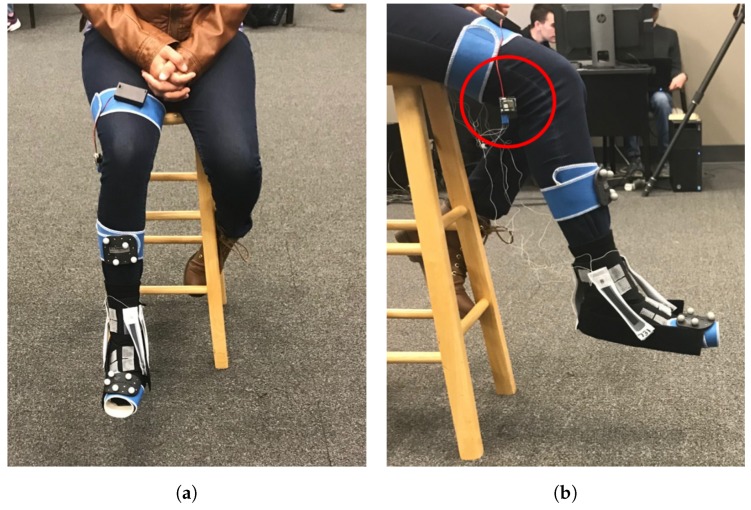
Participant image showing SRS and motion capture sensors: (**a**) front view; and (**b**) side view. The SRS sensing module is circled in red.

The participant was first instructed to fill out a participation consent form as per IRB protocol. The participant was then given a sock attached with Velcro mounts to place on their right foot. Participants were given either a small/medium or large/extra-large sock depending on their shoe size. Following this, the participant assumed a high-sitting position in an NWB position. For the familiarization process, the examiner aided the participant in a demonstration on how to perform each of the foot–ankle movements, encouraging the participant to perform full ROM (i.e., moving the foot as far as possible). Following this, the motion capture cluster sensors were mounted to the right foot and right shank, and the sensors were calibrated (see Figure 6).

A validation step was then conducted, where, in the NWB position, the participant performed full ROM of the ankle three times repeatedly, moving the foot in the specified direction, and then returning to its original position. Each trial began with the participant staged in a neutral position of the ankle (approximately zero degrees of DF/PF and zero degrees of INV/EVR) which was set by the examiner using a mechanical goniometer. This process was equivalent to one trial and was repeated for each of the four foot–ankle movements (plantar flexion, dorsiflexion, inversion and eversion).

Following the validation step, the SRS were mounted to the sock in POC 1, and the four movements were repeated. An example of a participant completing the trial with the sensors in POC 1 can be seen in Figure 6. This process was repeated for POC 2 and POC 3 (noting that the DF movement only had one POC). Both kinematic 3D motion capture data and raw capacitance data from the SRS were collected simultaneously for each of the sensor placement trials. Completion of the movement trials for the last SRS POC marked the completion of the testing procedures.

### 2.6. Data Analysis

The kinematic data of ankle ROM for each DOF were determined by using the Grood–Suntay angle orientation in the MotionMonitor^TM^ (Innovative Sports Training, Inc., Chicago, IL, USA) software. Raw kinematic data were filtered with a low-pass third-order Butterworth filter with a cut-off frequency of 15 Hz. The raw capacitance values of the SRS were measured using the 10 Channel SPI Sensing Circuit in conjunction with the Bluetooth Low Energy (BLE) module, both made by StretchSense. The values were recorded using the proprietary StretchSense BLE iOS application.

### 2.7. Statistical Analysis

The R language was chosen for formatting and analyzing the dataset, as R automated the analysis and is capable of powerful data visualization. The data for each trial was saved to a file that was named according to participant ID, SRS POC, and movement performed. This naming convention was used to pair each set of motion capture and SRS data files. When comparing data, the SRS positioned to measure plantar flexion and dorsiflexion were matched with the Flexion column recorded by MotionMonitor, and the SRS positioned to measure inversion and eversion were matched with the Inversion column recorded by MotionMonitor.

To compare the motion capture data to the SRS data, several preprocessing steps needed to be taken. First, the sampling rates needed to be matched between motion capture and SRS. Due to limitations of the StretchSense BLE module, the max sampling rate that could be used was 25 Hz, and after further processing through the StretchSense proprietary iOS application, the intervals between timestamps were not always consistent. To mitigate this, an R approximation function was used to interpolate the SRS values based on the timestamps collected from MotionMonitor, bringing the StretchSense data up to 100 Hz.

After upscaling the StretchSense SRS output, the data needed to be aligned over time with the motion capture output, as a slight delay existed due to the need to manually start the data recording on each measurement system. Cross correlation was utilized to determine the proper data time alignment, based on the recommendation of Rhudy [10]. The function was utilized based on the sensors measuring the movement performed (i.e., PF SRS used to align plantar flexion movement data), as it would be expected that both datasets would consistently produce three distinct peaks, each peak being representative of the foot movement performed by the participant during each trial. When EVR or PF was being measured, the motion capture data were inverted before performing cross correlation, as these movements were measured in the negative plane when recorded by MotionMonitor.

**Figure 7:**
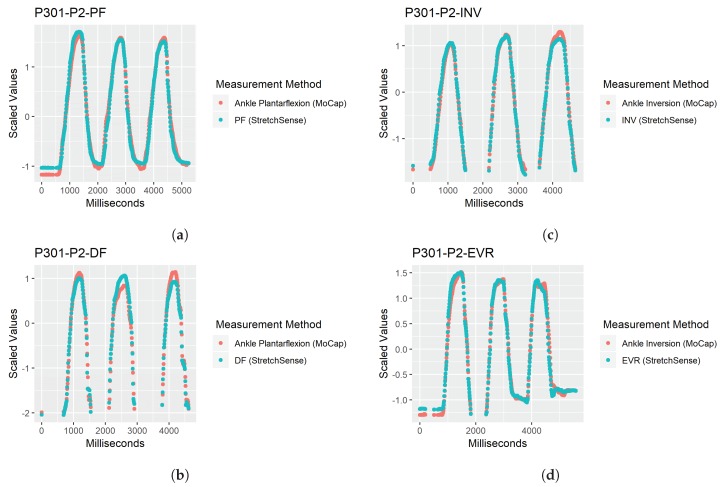
Examples of Preprocessed data: (**a**) preprocessed data for plantar flexion (PF) movement at POC 2; (**b**) preprocessed data for dorsiflexion (DF) movement at POC 2; (**c**) preprocessed data for inversion (INV) movement at POC 2; and (**d**) preprocessed data for eversion (EVR) movement at POC 2.

Finally, all data points where the motion capture data moved into a negative position or below the trial’s starting angle for the participant were dropped. This was implemented due to the sensor’s limitation of only being able to capture one direction of the ROM, since the sensor needs to be in tension. For example, if the participant was performing PF but went into slight DF during their foot–ankle movement, the sensor measuring PF would lose tension and record no movement, thus becoming unreliable for measuring a linear change in capacitance. The motion capture Flexion output would record this as a negative flexion value (indicating the participant moved into dorsiflexion), so these flexion values would be removed. Since the participant began each trial in a position of 0 degrees of flexion, this could be used as a reliable threshold for measurement, as care was taken to ensure there was some tension in the SRS at this position. An example of the preprocessed data is depicted in Figure 7. This graph presents scaled versions of the two datasets and was generated for all trials for visual inspection to make sure the data was preprocessed correctly.

**Figure 8:**
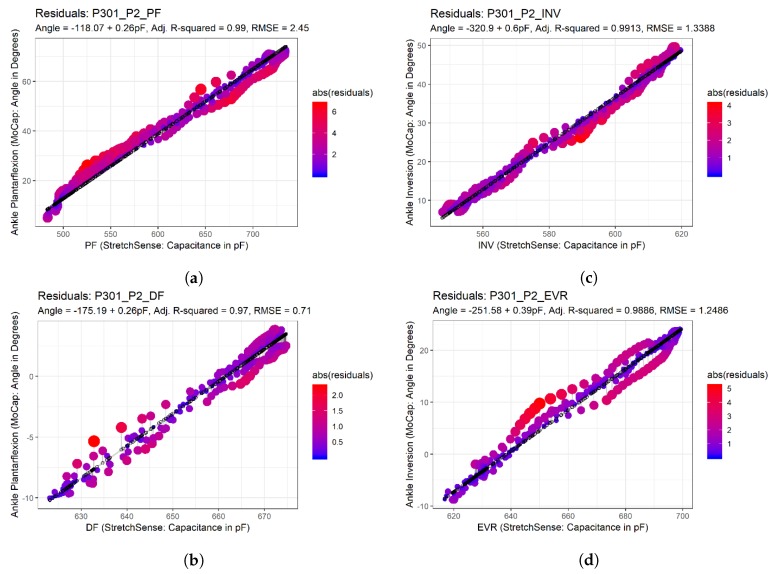
Residual plots: (**a**) residual plot for Plantar flexion movement at POC 2; (**b**) residual plot for Dorsiflexion movement at POC 2; (**c**) residual plot for Inversion movement at POC 2; and (**d**) residual plot for Eversion movement at POC 2.

Once the data were properly formatted, a linear model was created based on sensor capacitance versus motion capture angle. The $R^{2}$ value and root-mean-squared error (RMSE) were then calculated based on the linear model, and a plot of all residual values from the model was created for visual inspection. Figure 8 provides examples of this plot, with magnitudes of residuals being double encoded with size and color. Finally, a table was generated, characterizing each of the trials with the calculated $R^{2}$ value and RMSE to provide a measure of how well the SRS modeled the motion capture data.

To determine which sensor position configuration performed well at modeling the motion capture data, three metrics were considered: accuracy of angle prediction, consistency in prediction performance, and robustness across participants. Accuracy of angle prediction was determined based on observing which trials resulted in the highest average $R^{2}$ value, which indicates a goodness-of-fit of the linear model, and the lowest average RMSE value, which measures how accurately the model predicts the response in units of degrees [11]. Consistency in prediction performance was evaluated based on the standard deviation of the $R^{2}$ and RMSE values across all participants for each SRS POC.

## 3. Results

**Figure 9:**
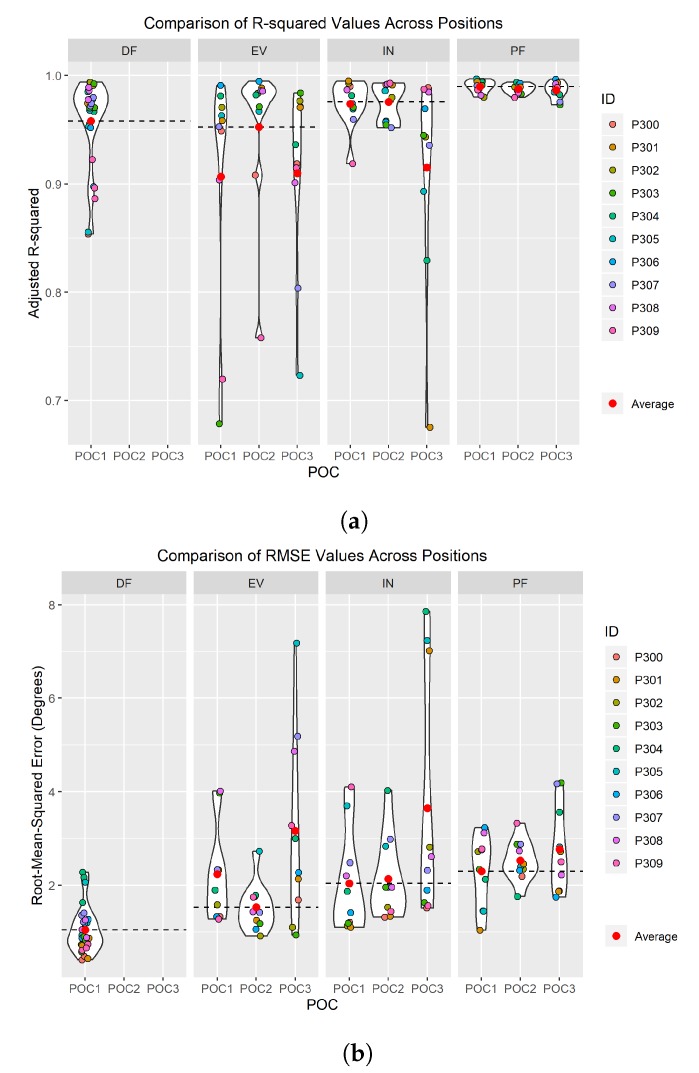
Violin plots: (**a**) *R*^2^ average and Violin plot comparison for all POCs; and (**b**) RMSE average and Violin plot comparison for all POCs.

The results for the three aforementioned metrics are summarized in Table 1. The best results for each metric for each POC are identified in bold. Figure 9 captures the performance of the accuracy and consistency metrics for each POC. The average values are calculated and plotted as single points, and a horizontal line is drawn to indicate the location of the best performing average value. Figure 9 also depicts violin plots, which are the curved areas along each POC that gives an idea of the “spread” of the data. These violin plots represent a kernel density distribution portrayed vertically. A greater horizontal width of a curve in the plot indicates a greater portion of participants that produced results near the value on the *y*-axis. For example, in Figure 9a, a large portion of participants produced an $R^{2}$ around 0.95–0.99 for the INV sensor at POC 2, while participants were more spread out at $R^{2}$ values of 0.97–0.65 for the INV sensor at POC 3. When comparing these graphs visually to the standard deviation values in Table 1, distributions resulting in the lowest standard deviation values are easily identifiable. Additionally, given that the individual participant data points reiterate the “spread” of the data, the POCs producing the most outliers can be easily identified as well.

**Figure 10:**
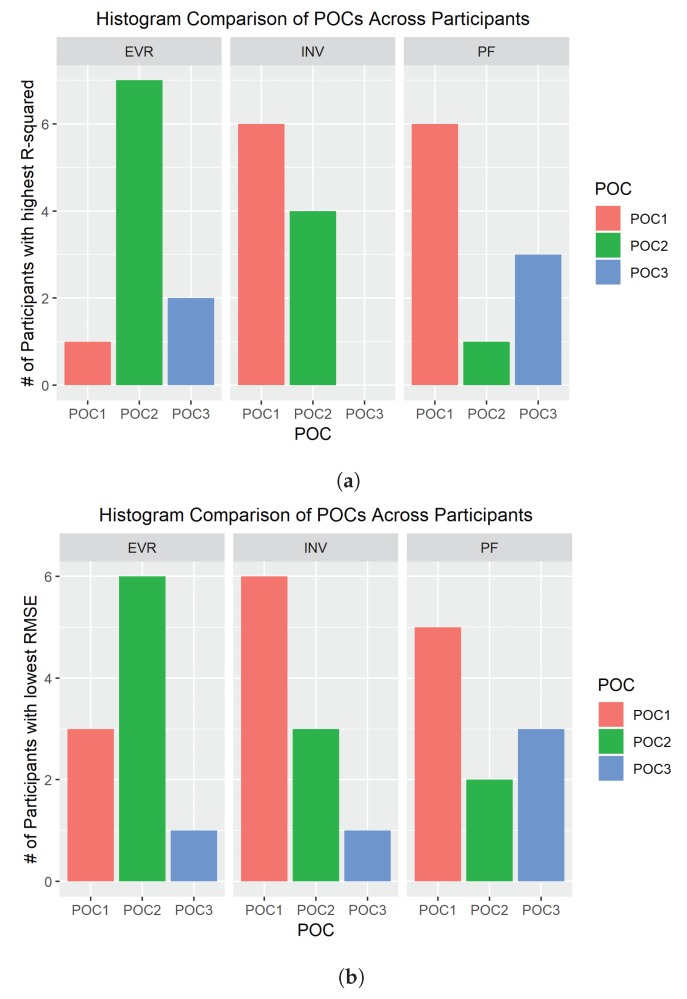
Number of participant counts: (**a**) number of participants where SRS POC resulted in the highest *R*^2^ values; and (**b**) number of participants where SRS POC resulted in the lowest RMSE value.

To determine which SRS POCs were the most robust, a histogram was created based on which POC produced the highest $R^{2}$ value or lowest RMSE value for each participant. The POC that most frequently resulted in the best value was considered the most robust. The motivation for this metric was to get an idea for how well each POC performed against the variety of foot sizes amongst each of the participants. The results of this metric are seen in Figure 10 and also recorded in Table 1.

The SRS were also evaluated to determine how well they tracked multiple movements beyond the single movement that they were positioned to record. For example, the PF SRS is placed on the top of the foot–ankle to capture the plantar flexion movement, but because the participants also performed INV and EVR movements while the PF SRS was mounted, the PF SRS could be evaluated for other foot–ankle movements; essentially, this assessment was completed to determine if measuring multiple movements with one SRS was feasible. Thus, a new table was generated identifying $R^{2}$ and RMSE values for all four SRS for each trial, regardless of the primary movement that was being measured.

**Figure 11:**
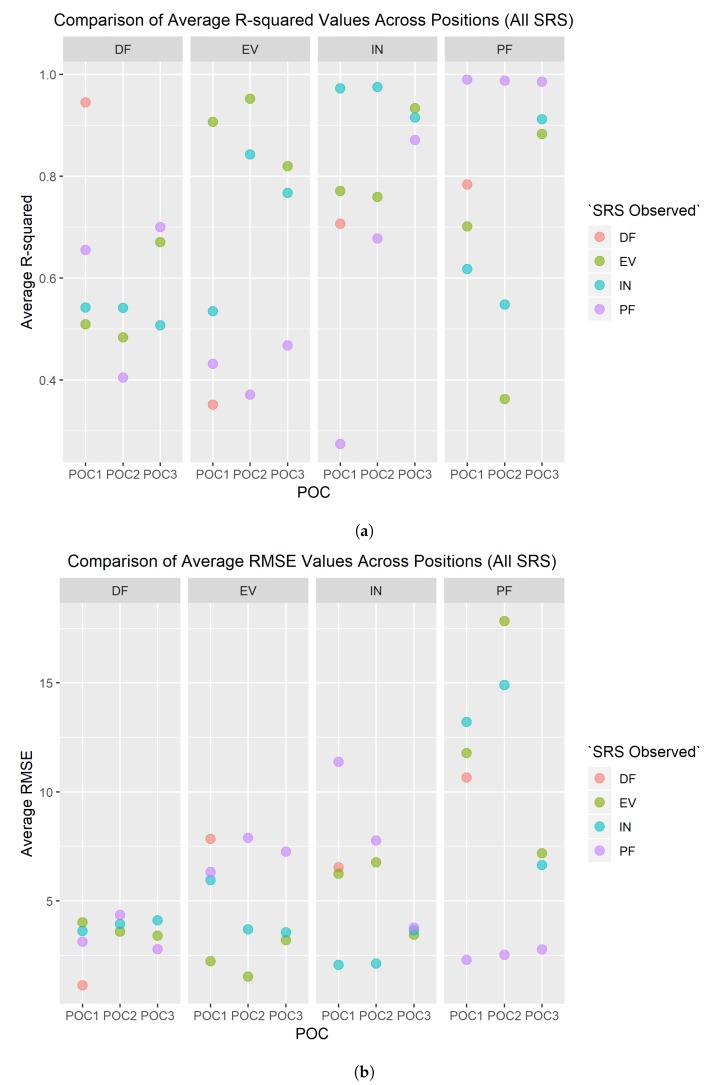
Comparison of *R*^2^ and RMSE values: (**a**) comparison of average *R*^2^ values for all SRS at all POCs; and (**b**) comparison of average RMSE values for all SRS at all POCs. Note that in both comparisons DF only had one SRS POC.

In Figure 11, the results can be seen for each movement based on the SRS that was observed. The titles for each subgraph indicate the movement that was performed by the participant, while the color of each data point represents the movement that the SRS was originally intended to measure based on the POC. One thing to note on the $R^{2}$ graph is that an inverse relationship will also produce a high $R^{2}$ value, hence why the EVR SRS produced a reasonably good $R^{2}$ value when used to measure the INV movement. As the foot goes further in the INV movement, it can be expected that the EVR SRS would decrease in value, if there is still tension in the SRS.

**Figure 12:**
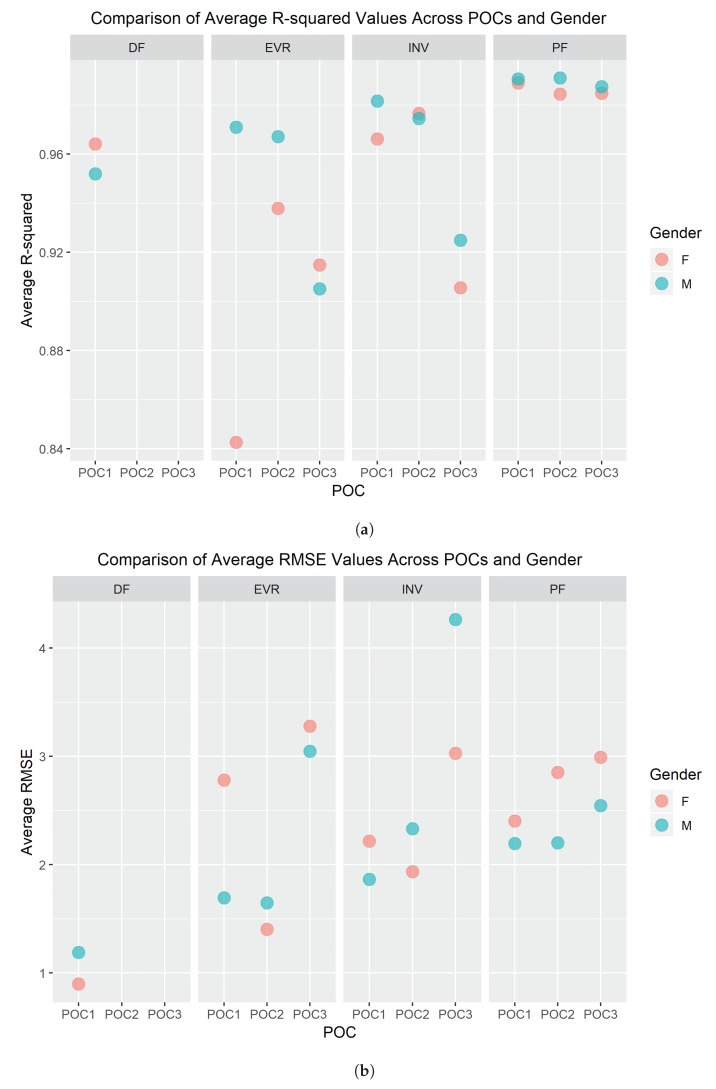
(**a**) Comparison of average *R*^2^ values for all SRS between genders/foot size; and (**b**) comparison of average RMSE values for all SRS between genders/foot size.

Lastly, gender and shoe size were compared to see if they influenced the quality of the SRS’s measurements. For this study, all females used the small/medium sock, while all males used the large/extra-large socks, so both categories are encoded in the following results. Figure 12 presents average $R^{2}$ and RMSE values between both genders for each SRS POC.

## 4. Discussion

The purpose of the study was to use a 3D motion capture system of human participants to identify SRS POCs that accurately record the four primary movements of the foot–ankle complex: PF, DF, INV, and EVR. More specifically, the purpose was to examine what combination of four SRS will most accurately measure ankle movements in four directions compared to the golden standard for human movement assessment. Given the complex nature of using a linear solution to accurately quantify movement of a triaxial joint, movement was captured from 10 participants and multiple tests were performed to analyze the SRS capacitance output from multiple positions around the foot–ankle. This section outlines the findings for each of the four individual movements analyzed in this study from most simple POC placement preference to the most difficult.

### 4.1. Dorsiflexion (DF)

DF was the only foot–ankle movement where just one POC placement was tested. Only one POC was needed for this movement because the anatomy on the posterior side of the foot, primarily the calcaneus (heel), only provides one location for POC to accurately measure dorsiflexion [7,8]. However, SRS accuracy still needed to be validated against motion capture similarly to how all other SRS POCs were evaluated in order to establish baseline confidence in the solution. DF POC 1 had a high $R^{2}$ average above 0.95 over all participants meaning that nearly all values of the SRS correlate directly to the flexion values captured via motion capture (Table 1 and Figure 9a). Therefore, there is a high level of fit between SRS and motion capture for the DF movement across all participants. Likewise, where a high $R^{2}$ average is an indicator of relative measure of fit, a low RMSE average value indicates an absolute measure of fit. DF POC 1 scored a low value for all participants for RMSE average further confirming the SRS measurement’s fit to motion capture (Figure 9b). PF was the closest comparable movement as Figure 11 demonstrates that DF and PF are opposing movements [7]. POC 1 was confirmed to be the proper SRS placement for DF and there was minimal difference in average $R^{2}$ and RMSE values between men and women and/or foot size (Figure 12).

### 4.2. Eversion (EVR)

The second simplest SRS placement was found to be POC 2 for EVR. Three POCs were tested and six measurements were taken to decide optimal positioning for EVR. All six measurements (Table 1) clearly indicate POC 2 as the best location for SRS based data collected via motion capture. The $R^{2}$ average was high, over 0.95, and RMSE average was low indicating good fits for both relative and absolute measures respectively (Figure 9). Most participants had the highest $R^{2}$ values and lowest RMSE values for POC 2. POC 2 demonstrated the best comparison movement with INV (Figure 11), the proper opposing movement to EVR, for both $R^{2}$ and RMSE calculations. The comparison of $R^{2}$ and RMSE for EVR between men and women participants occurred at POC 2 as well, as observed in Figure 12.

**Figure 13:**
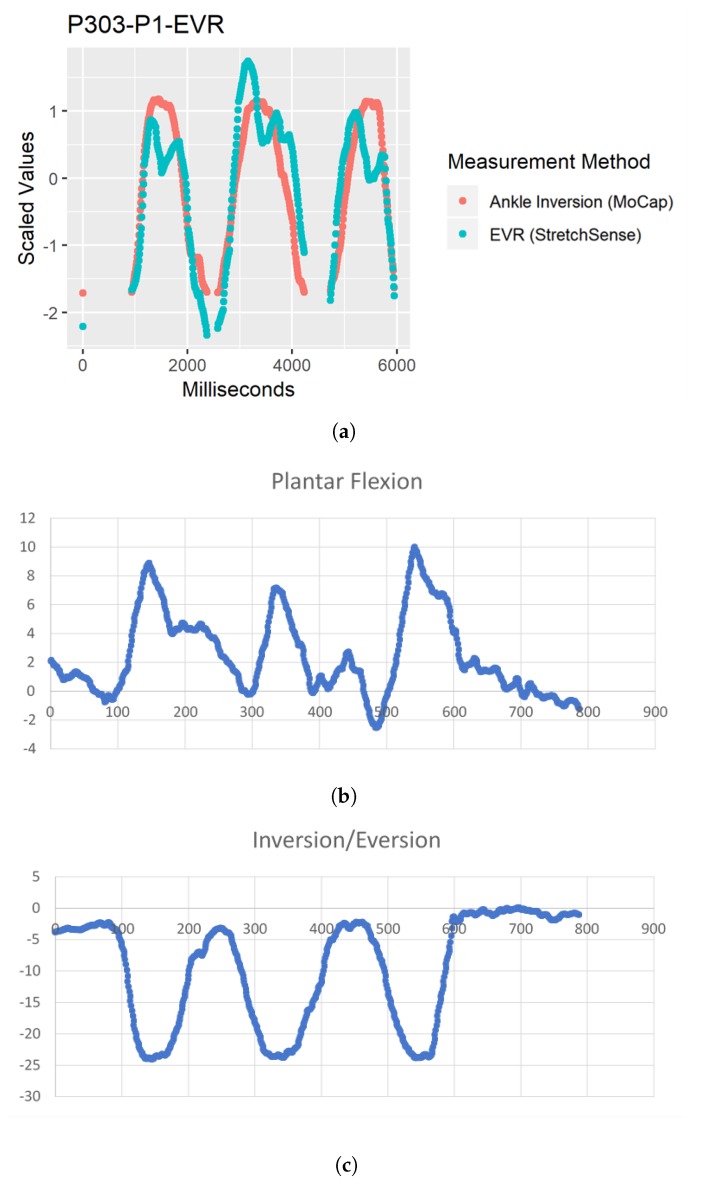
(**a**) Comparison of motion capture and StretchSense when EVR movement not performed in isolation. Both datasets scaled and motion capture data inverted to depict relationship between sensing methods. (**b**) Raw motion capture data for PF movement. (**c**) Raw motion capture data for EVR movement (negative values).

While no outliers were removed for this study, there were some notably different data points for EVR $R^{2}$ calculations and EVR POC 3 RMSE that needed to be investigated to ensure that POC 2 is absolutely the better SRS placement selection. For a few of the participants, performing foot–ankle movements such as EVR and INV in isolation was difficult because some PF also occurs with INV and EVR movements [7,8,12,13,14]. The worst case of a participant’s inability to disassociate the two movements and perform true INV and EVR movements was captured by participant P03. To confirm that P03’s movement should not impact final placement decisions for EVR, the motion capture data were evaluated to understand why the participant’s $R^{2}$ and RMSE averages were much lower and higher, respectively. Figure 13 demonstrates that, as P03’s “Inversion/Eversion” movement experienced negative peaks (indicating an EVR movement), their “Plantar flexion” movement peaked showing a correlation between the two movements and thereby confirming that EVR for P03 was not in true isolation. The reason this is important from an SRS placement perspective is that, by moving into PF, P03 reduced tension on the SRS and therefore reduced the capacitance value output indicating that the SRS output would not demonstrate as strong of an EVR movement as compared to the true EVR movement captured in motion capture, thus further weakening $R^{2}$ and RMSE fit.

### 4.3. Plantar Flexion (PF)

SRS placement for PF was not quite as definitive as EVR but was still very compelling as four of six metrics used for evaluation Figure 3 indicated POC 1 being the best fit. $R^{2}$ average value was extremely high at over 0.98 and RMSE average was the lowest of the three positions for POC 1 (Figure 9). While standard deviation of $R^{2}$ and RMSE was not the lowest for POC 1, six of 10 participants had the highest $R^{2}$ and lowest RMSE for this position (Figure 10). DF is the closest opposing movement for POC 1 (Figure 12) as expected while POC 1 indicates the best fit for both men and women (Figure 13).

### 4.4. Inversion (INV)

Of all the movements, determining the placement for INV was the most challenging, because two POC options, POC 1 and POC 2, appeared to be nearly identical in performance. POC 2 demonstrated the best $R^{2}$ average at over 0.97 (Figure 9a), but POC 1 was just slightly lower (Table 1). RMSE average was lowest for POC 1 (Figure 9b), but POC 2 was only slightly higher. Standard deviation adjustments for $R^{2}$ and RMSE both indicated POC 2 as the better fit but the total number of participants with the better fit values indicated POC 1 as the optimal location for placement (Figure 10). To make a decision as to the best POC for SRS placement regarding INV movements, the research team considered the following three pieces of information: (a) $R^{2}$ is often viewed to be the better indicator of fit; (b) the POC selected for EVR (the opposing movement to INV) in order to promote uniformity and sensor placement during wearable design; and (c) picking either POC has limited negative ramifications given that the output for each is so similar. For this reason, POC 2 was selected for INV. these results indicate that both POC 1 and POC 2 provided good results, and that this motion was not as clearly delineated as the other movement cases.

**Figure 14:**
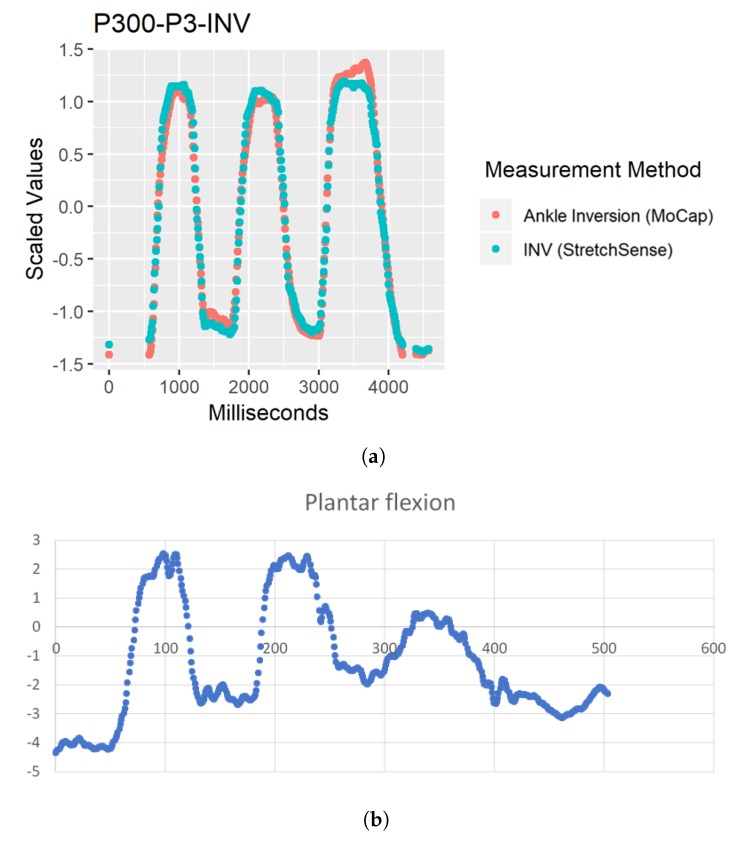

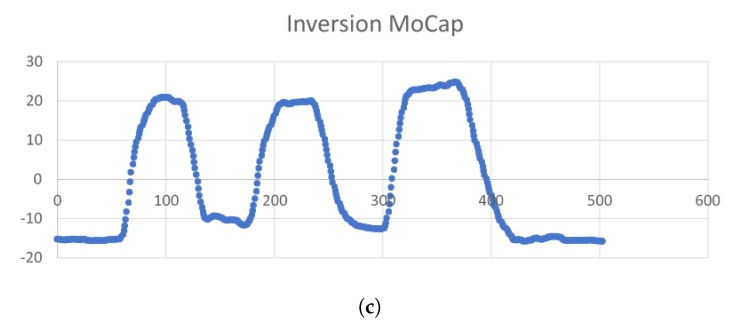
(**a**) Comparison of motion capture and StretchSense when INV movement not performed in isolation. Both datasets scaled to depict relationship between sensing methods. (**b**) Raw motion capture data for PF movement. (**c**) Raw motion capture data for INV movement (positive values).

Similar to EVR, some participants had issues isolating their movements to only perform INV without PF. Inversion is a difficult joint action to perform in isolation [7,8,12,13,14]. In addition, similar to Figure 13, Figure 14 shows that, as their “Inversion MoCap” movement experienced positive peaks (indicating an INV movement), their plantar flexion movement also peaked showing the same correlation between the two movements while also reducing stretch on the SRS thereby reducing capacitance value output.

### 4.5. Limitations

As noted in the Discussion Section, a few participants had a difficult time isolating their movements and so PF was performed during INV and EVR trials. This is not an issue for motion capture as the cameras and markers can accurately observe all joint angle movements regardless of the movement type. However, SRS capacitance values are dependent upon consistent tension and stretch that occurs linearly. By performing two movements at once (e.g., PF and INV), the tension was reduced, and capacitance was lowered thus recording “weaker” values and further lowering and raising $R^{2}$ and RMSE averages, respectively. This was only an issue, however, because one sensor was being observed at a time. With multiple SRS output being captured simultaneously for all four movements, PF would have been captured in addition to INV and EVR. However, this does not solve the issue of a lack of tension in the INV and EVR SRS every time an additional PF movement is performed. Mitigating these issues will likely require additional investigation into the initial “tightness” of the SRS while the wearer is in a neutral position. In addition, because EVR and INV are opposing movements, information about one movement can be inferred from the other. Further, with all four sensors collecting foot–ankle movement data, machine learning solutions can be developed to further enhance robustness of the solution. Utilizing more sophisticated modeling techniques with multiple sensors as inputs, the output angles for each foot–ankle complex motion may become more robust and have lower errors.

In addition, this study used participants with bare feet (i.e., no shoes). From a product development perspective, creating a solution that utilized the capabilities of the SRS while still allowing the users to wear shoes would be the ideal scenario. The intent of this study was to find the best SRS placements in order to accurately quantify foot–ankle movements. While a sensor that conforms to the curvature of the ankle complex on top of the sock is an ideal end state, this study was strictly about validating placement. Creating a fully SRS-integrated, sensor-ladened compression sock is the end goal for this research, but confidence in positioning is necessary prior to incorporation of the technology into the textile materials.

### 4.6. Future Work

With each SRS POC identified, the next logical step is to conduct a study where all four SRS are placed on participants who are performing dynamic movements such as walking while also wearing motion capture markers for continued validation. With walking data, the research team will begin to understand more about the interactions between all four sensors as they collect information on the same movement but from different locations about the foot and ankle. The same validation tests can be performed as an assessment of fit that will be performed against motion capture output. Basic walking movements on a flat surface at a normal pace will need to be observed. In addition, to engage true INV and EVR movements, participants will need to walk on a slightly inclined surface. Further, both feet and ankles will need to be assessed during the same gait movement. Should this SRS solution be verified as an accurate assessment tool for gait, there will be many rehabilitation- [15,16,17] and athletic-based [18,19] opportunities for an off-the-shelf sensor solution that nearly duplicates the accuracy of motion capture yet was originally intended for the use in soft robotics.

Machine learning has been applied to body sensor networks [20], rowing coaching [21], and human gait analysis [22,23], to name a few. We plan to apply machine learning techniques when linear models are not adequate or to combine multiple SRS inputs into an output, such as estimating INV or EVR angles.

Our current study is a preliminary work on testing the efficacy of the wearable sensors for the foot–ankle complex movements. In this regard, our focus was to assess the performance of the sensors within the first standard deviation, and, hence, the sample size of ten human subjects. However, in considering the design of wearable sensors for the foot–ankle movements, we plan to test the performance of these wearable sensors that would be applicable for 95% of human subjects (the human subject number would be greater than that implemented in the current study).

## 5. Conclusions

Upon completion of the participant trials, $R^{2}$ and RMSE averages were used to determine both relative and absolute fit for the SRS output as compared to motion capture. Robustness was determined by how many participants had the same POC selection for each movement type. Standard deviation for $R^{2}$ and RMSE was calculated for each participant as well. In addition, opposing movement comparisons were made for each SRS POC and comparisons between gender and foot size were observed.

Through these many evaluations, SRS POCs that best captured foot–ankle movement were determined with a high level of confidence. POC 1 was selected for both PF and DF and POC 2 was selected for both INV and EVR. With these placements identified, this research team is confident that a true gait assessment that rivals the accuracy of 3D motion capture systems can be performed using only four, off-the-shelf SRS sensors. The solution verified on human participants within this study essentially creates a real-time, continuous, and consistent electric goniometer that a user can wear to accurately assess their movements “from the ground up” [3].

## Figures and Tables

**Table 1:** Averages, standard deviations, and participant count of *R*^2^ and RMSE values for all POCs; best POC in bold. * Denotes preferred POC location for each of the four foot–ankle movements. Deg., degrees; Std. Dev., standard deviation.

Movement	Position and Orientation Configuration (POC)	*R*^2^ Average (Figure 9a)	RMSE Average (deg.) (Figure 9b)	*R*^2^ Std. Dev.	RMSE Std. Dev. (deg.)	Higest *R*^2^ Number of Participants (Figure 10a)	Lowest RMSE Number of Participants (Figure 10b)
EVR	POC 1	0.9068	2.2378	0.1123	1.0159	1	3
EVR	POC 2 *	**0.9525**	**1.5269**	**0.0727**	**0.5186**	**7**	**6**
EVR	POC 3	0.9100	3.1628	0.0845	2.0127	2	1
INV	POC 1	0.9739	**2.0407**	0.0225	1.0939	**6**	**6**
INV	POC 2 *	**0.9755**	2.1340	**0.0178**	**0.8838**	4	3
INV	POC 3	0.9153	3.6455	0.0977	2.6128	0	1
PF	POC 1 *	**0.9898**	**2.2996**	0.0056	0.7616	**6**	**5**
PF	POC 2	0.9877	2.5274	**0.0046**	**0.4376**	1	2
PF	POC 3	0.9861	2.7679	0.0076	0.9216	3	3
DF	POC 1 *	0.9567	1.0568	0.0410	0.4952	N/A	N/A
